# A ML Approach for MACEs Prediction in Patients With UA and HFpEF

**DOI:** 10.1093/geroni/igaf122.3518

**Published:** 2025-12-31

**Authors:** Yijun Wang, Jinhui Wu

**Affiliations:** West ChinaHospital, Sichuan University, Chengdu, Sichuan, China (People’s Republic); West ChinaHospital, Sichuan University, Chengdu, Sichuan, China (People’s Republic)

## Abstract

Background Heart failure with preserved ejection fraction (HFpEF) and unstable angina (UA) often coexist in clinical practice, constituting a high-risk cardiovascular phenotype with a markedly increased incidence of major adverse cardiovascular events (MACEs). Objective This study aimed to develop models based on machine learning (ML) algorithms to estimate the risk of MACEs in patients with coexisting UA and HFpEF. Methods This cohort study included 2,638 patients with both HFpEF and UA between January 1, 2015, and December 31, 2021. Patients were divided into the derivation cohort (n = 1,367) and validation cohort (n = 1,271) based on geographic regions. Clinical, laboratory, and imaging data were extracted from electronic medical records. Key predictors were identified using a hybrid feature selection method combining LASSO and Boruta algorithms. Nine ML models were developed: XGBoost, LGBM, RF, DT, GBDT, KNN, SVM, MLP, and GNB. Model performance was evaluated for the area under the receiver operating characteristic curve, accuracy, sensitivity, specificity, recall, F1-score, precision-recall curve, Brier scores, calibration curves and decision curve analysis. The best-performing model was deployed as a web-based risk calculator. Results Using a combination of LASSO regression and the Boruta algorithm, seven key predictors were identified: Diabetes Mellitus, TG/HDL-C ratio, SIRI, TyG-BMI index, Creatinine, NT-proBNP, and Gensini score. The XGBoost-based model exhibited superior performance with an AUC of 0.825 in the derivation cohort and an AUC of 0.781 in the validation cohort based on seven features. Conclusion XGBoost-based predictive model constitutes an innovative approach to risk stratification for the complex dual phenotype of HFpEF and UA.

